# Meta-analysis and machine learning-augmented mixed effects cohort analysis of improved diets among 5847 medical trainees, providers and patients

**DOI:** 10.1017/S1368980021002809

**Published:** 2022-02

**Authors:** Dominique J Monlezun, Christopher Carr, Tianhua Niu, Francesco Nordio, Nicole DeValle, Leah Sarris, Timothy Harlan

**Affiliations:** 1 Department of Cardiology, The University of Texas MD Anderson Cancer Center, 1400 Pressler Street, Unit 1451, Houston, TX 77030, USA; 2 Center for Artificial Intelligence & Health Equity, Global System Analytics & Structures, New Orleans, LA, USA; 3 The Goldring Center for Culinary Medicine, Tulane University, School of Medicine, New Orleans, LA, USA; 4 Department of Biochemistry and Molecular Biology, Tulane University, School of Medicine, New Orleans, LA, USA; 5 Department of Medicine, Brigham and Women’s Hospital, Harvard University, Boston, MA, USA; 6 GWU Culinary Medicine Program, George Washington School of Medicine & Health Sciences, Washington, DC, USA

**Keywords:** Nutrition education, Meta-analysis, Obesity, CVD, Machine learning

## Abstract

**Objective:**

We sought to produce the first meta-analysis (of medical trainee competency improvement in nutrition counseling) informing the first cohort study of patient diet improvement through medical trainees and providers counseling patients on nutrition.

**Design:**

(Part A) A systematic review and meta-analysis informing (Part B) the intervention analysed in the world’s largest prospective multi-centre cohort study on hands-on cooking and nutrition education for medical trainees, providers and patients.

**Settings:**

(A) Medical educational institutions. (B) Teaching kitchens.

**Participants:**

(A) Medical trainees. (B) Trainees, providers and patients.

**Results:**

(A) Of the 212 citations identified (*n* 1698 trainees), eleven studies met inclusion criteria. The overall effect size was 9·80 (95 % CI (7·15, 12·45) and 95 % CI (6·87, 13·85); *P* < 0·001), comparable with the machine learning (ML)-augmented results. The number needed to treat for the top performing high-quality study was 12. (B) The hands-on cooking and nutrition education curriculum from the top performing study were applied for medical trainees and providers who subsequently taught patients in the same curriculum (*n* 5847). The intervention compared with standard medical care and education alone significantly increased the odds of superior diets (high/medium *v*. low Mediterranean diet adherence) for residents/fellows most (OR 10·79, 95 % CI (4·94, 23·58); *P* < 0·001) followed by students (OR 9·62, 95 % CI (5·92, 15·63); *P* < 0·001), providers (OR 5·19, 95 % CI (3·23, 8·32), *P* < 0·001) and patients (OR 2·48, 95 % CI (1·38, 4·45); *P* = 0·002), results consistent with those from ML.

**Conclusions:**

The current study suggests that medical trainees and providers can improve patients’ diets with nutrition counseling in a manner that is clinically and cost effective and may simultaneously advance societal equity.

The global obesity epidemic and its related chronic comorbidities including CVD remain the world’s top morbidity causes, with CVD alone accounting for over one in three deaths, despite the clinically effective, cost efficient and societally equitable role of nutrition intervention at reducing the staggering toll of this epidemic^(1–5)^. Yet, it has outpaced medical education’s response^(6)^. Although a recent *JAMA* study identifies diet as a top morbidity and mortality risk factor^(7)^, only a minority of primary care physicians regularly counsel their patients in nutrition or monitor their BMI^(8)^. A central cause appears to be training deficiencies^(9)^, as approximately 50 % of paediatricians and internists have inadequate competencies for educating patients on obesity and even basic nutrition^(10)^. This lack of proficiency appears to stretch back to medical education as three in four medical schools fail to achieve the minimum 25 h of diet training as outlined by the National Academy of Sciences^(11)^. Accordingly, 81 % of medical students by the time of graduation report they are inadequately equipped to counsel patients on nutrition^(12)^. Increasing evidence underscores the necessity of comprehensive interdisciplinary obesity and nutrition training in medical schools^(13,14)^. But amid the growing push among medical professionals for improved training^(14)^, recent attempts to improve this education through medical schools and residency programmes share several significant limitations that reduce their suitability to contribute to improved training.

Past studies assessing the effectiveness of nutrition education interventions lacked: control groups^(15–22)^, validated surveys^(14,15,19,23)^, longitudinal follow-up^(14,16,17,19–21)^, deliberate practice educating patients^(15,17–22)^, adequate sample sizes^(14–18,20–22)^, causal inference analysis^(15–23)^ and the Mediterranean diet (MD)^(14–22)^. Though one study (Schlair *et al.* 2012) unlike the others attempted to control for baseline selection bias through multivariable regression, no studies utilised study design or analysis methodology to allow causal inference. Since these studies also fail to feature the most extensively supported diet for patients, the MD^(24–27)^, best practices in nutrition counseling are further limited.

Recent studies additionally omitted two key trends in evidence-based medical education: simulation-based medical education with deliberate practice and comparative effectiveness research. Robust meta-analysis evidence indicates that actively implementing new knowledge and skills produces superior mastery over passively learning material through lectures or even through team learning^(28)^. These studies also fall short of adhering to the comparative effectiveness research recommendations of the Institute of Medicine attempting to improve research methodologies and thus study validity^(29)^. Instead of featuring the comparative effectiveness research prioritisation of testing one treatment effectiveness over another, these recent nutrition studies simply deal with treatment efficacy and thus cannot answer whether the current treatment is superior to prior. Finally, these prior studies are largely silent on two top comparative effectiveness research components of social disparities and healthcare systems, within which treatments are nested and by which they are largely influenced. The paucity of studies with robust programme and methodology strengths thus reduce the evidence-base available to derive effective, equitable health policy on nutrition curriculum guidelines and dissemination of effective models for public health and clinical applications. We therefore sought to produce the first known systematic review and meta-analysis of nutrition education interventions for medical student and resident improvement in their competencies counseling patients in nutrition (part A of this study). It then guided the largest machine learning (ML)-augmented multi-centre prospective cohort study on hands-on cooking and nutrition education to improve medical trainees and providers’ diets and competencies educating patients on nutrition, and thus patient diet and health outcomes (Cooking for Health Optimization for Patients (CHOP), ClinicalTrials.gov NCT03443635) (part B of this study).

## Materials and methods

### Part A: systematic review and meta-analysis

#### Systematic search

Papers were considered for inclusion in the current study by first identifying them in the Embase, PubMed and Web of Science databases from January 1, 1994, to March 31, 2018. Papers were located based on pre-defined search terms: diet education, nutrition education, curriculum, classes, modules, online, learning, problem-based learning, medical schools, medical students, students, residents, physicians, medical professionals and health professionals. Author discussion resolved disagreements. The Preferred Reporting Items for Systematic Reviews and Meta-Analyses guideline was the standard by which the current study was conducted^(30)^.

#### Inclusion and exclusion criteria

The inclusion criteria were applied after detailed consideration of the limited research in this field and the even more pronounced absence of widely accepted treatment models, endpoints and analytic techniques. Studies were included in this systematic review and meta-analysis if they used a pre-post design, tested a nutrition education as treatment, included the outcomes of trainee competencies counseling patients on nutrition and was performed in the 25 years prior to this meta-analysis. Pre-post studies were included given the prevalent absence in the studies of a control group. The date range was selected based on the medical school reforms nationally enacted in 1995 onward that subsequently impacted trainee programmes globally. Studies were excluded if they omitted adequate information on treatment effect and s e, or if there were redundant publications or non-original reports (such as reviews, editorials or meta-analyses). The larger or more recent study by a particular author within the same case series was included as applicable.

#### Data coding information

The extraction was done by two reviewers separately with disagreement resolved through discussion. The primary outcome was trainee competency educating patients on nutrition. Pre-defined study parameters were identified from the studies meeting criteria through standard form, including study design, primary author, publication year, nation, sample size, time of follow-up and effect size (ES) with the associated 95 % CI.

#### Study quality scoring criteria

The quality of studies was assessed using the standard forty-point STROBE quality scale independently by two authors (DA and RS)^(31)^. Studies were categorised according to a level according to their quality scores: low (0–19), moderate (20–29) and high (30–40). Disagreements were resolved by author discussion.

#### Statistical methods

Mean competency differences were calculated between post and pretest percentages and then divided by the pretest s d to produce the standardised ES for each study. *T*-test values and df were utilised to calculate ES correlation for studies lacking adequate information to calculate the ES^(32)^. Correlation coefficients were produced by each study’s ES after it was corrected by the sample size as appropriate. The ML technique of random forest multiple imputation was applied to improve performance of the traditional statistical method of standard multiple imputation by chained equations given its demonstrated superior accuracy and efficiency than multiple imputation by chained equations alone^(33)^.

Inverse-variance weighted fixed or DerSimonian and Laird random effects meta-analysis models were initially considered to calculate the pooled estimates and 95 % CI of the correlations^(32)^. Random effects were ultimately selected if significant heterogeneity existed across the individual studies, which was assessed using the *I* ^2^ statistic ≥75 % Cochran’s *Q*-test (*P* < 0·100)^(34,35)^. These heterogeneity tests were included given the assumption of the current study that prior nutrition studies for medical trainees would have varying degrees of quality, follow-up times and education interventions^(32)^. Sub-group analysis by study quality was additionally done. Cochran’s *Q*-test and the *I* ^2^ statistic were utilised to compare the sub-group differences with significant heterogeneity detected using a *P*-value <0·10. Harbord–Egger and Begg–Mazumdar statistical tests were utilised to qualitatively and quantitatively assess publication bias, since this study’s overall ES across the different studies could be inappropriately skewed if studies with smaller p-values or larger sample sizes had greater publication chances^(36,37)^. Both techniques were used since heterogeneity across studies by virtue of their varying study parameters or quality could alone produce non-symmetrical funnel plots, since more symmetrical plots are created by larger ES if there are a greater number of studies which meet criteria for meta-analysis inclusion^(38,39)^. Publication bias would be controlled for through the Duval and Tweedie nonparametric ‘trim and fill’ method^(40)^. Rank-based data augmentation in this method accounts for studies absent from the published literature to produce estimates of the number of missing studies and the associated ES:

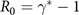




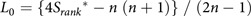




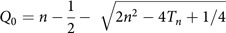

with 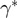 indicating the length of the rightmost rank runs, 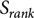 indicating the Wilcoxon statistic and *n* indicating the number of studies when the studies included in the meta-analysis are ranked according to their distance from the pooled effect^(40,41)^. This adjustment method was selected due to its accepted role within meta-analyses^(42)^, its efficient and consistent results^(43,44)^, its versatility for smaller data sets^(45)^, the less defined mathematical justification of its method competitors such as Copas and the corresponding absence of evidence indicating superiority of other methods to this one^(42)^. ML was then applied to test performance of the statistical meta-analysis results produced through fixed and random effects. The top algorithm was selected by lowest root relative squared error among the 26 ML algorithms appropriate for the ES continuous outcome, with values < 100 % being more favorably considered and then compared with the traditional meta-analysis results. All above analyses defined as traditional statistical analyses were performed using Stata 14.2 (StataCorp.), and ML analysis was performed with Java 9 (Oracle, Redwood Chores, California, USA)^(46)^. Except as otherwise specified, the significance level was defined at a two tailed *P*-value of 0·05.

### Part B: cohort study

In the second phase of this study, the top performing curriculum identified above was utilised in the CHOP study in which medical trainees and providers were first educated on nutrition in the first medical school-based teaching kitchen, Tulane University School of Medicine’s Goldring Center for Culinary Medicine (GCCM) and then they provided free cooking and nutrition classes for predominantly lower income patients. The meta-analysis was updated annually using the above methodology to inform whether modifications were required to the GCCM curriculum for the cohort study. Multilevel mixed effect multivariable regression was conducted on the 5847 subjects meeting CHOP study criteria from fall 2012–2018 (including consecutive medical trainees, medical providers and patients from the thirty-two participating health centres or universities nationally who voluntarily identified themselves to the above institutions and subsequently completed at least one pre-GCCM course validated survey^(47)^). Model performance was confirmed by standard regression diagnostics in addition to comparison to ML-based backward propagation neural networks using accuracy and root mean squared error. The primary outcome was high/medium *v*. low Mediterranean diet (MedDiet) adherence. High/medium adherence was set at 4–9 as adapted from the nine-point scale described in the seminal *New England Journal of Medicine* study by Trichipoulou *et al.* ^(24)^ and later utilised in the multi-centre randomised trial in the same journal by Estruch *et al.* ^(3)^

## Results

### Part A: systematic review and meta-analysis

**Fig. 1:**
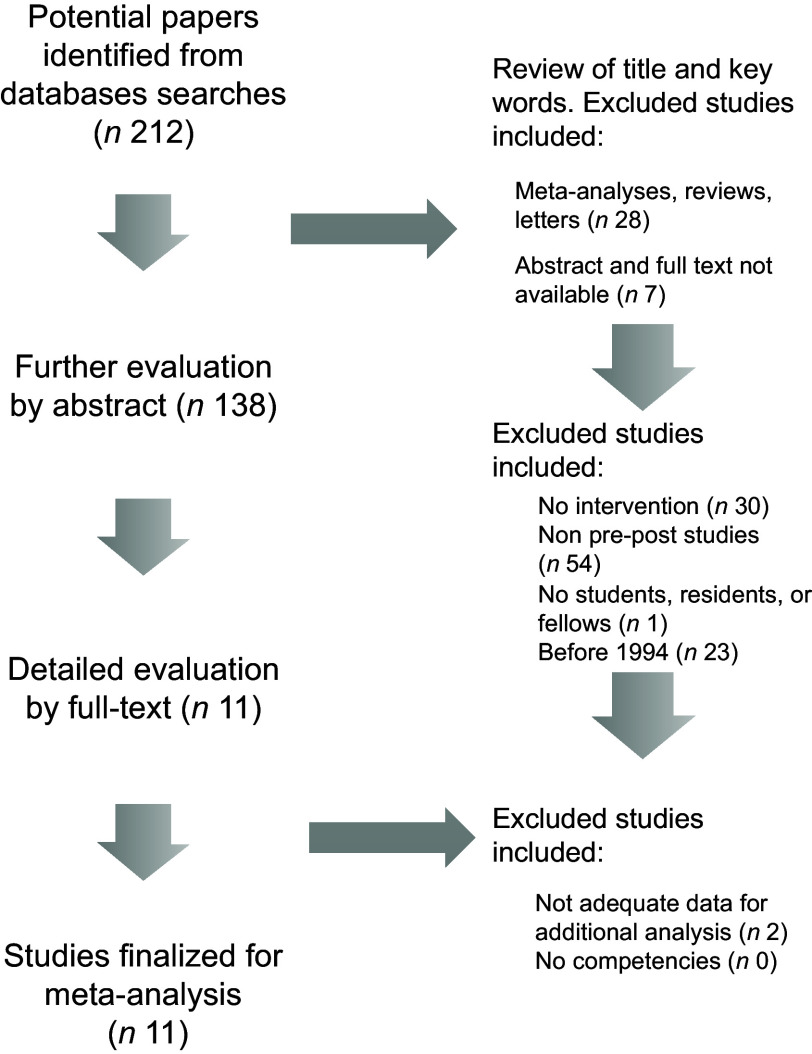
Flow chart for study data extraction

**Fig. 2:**
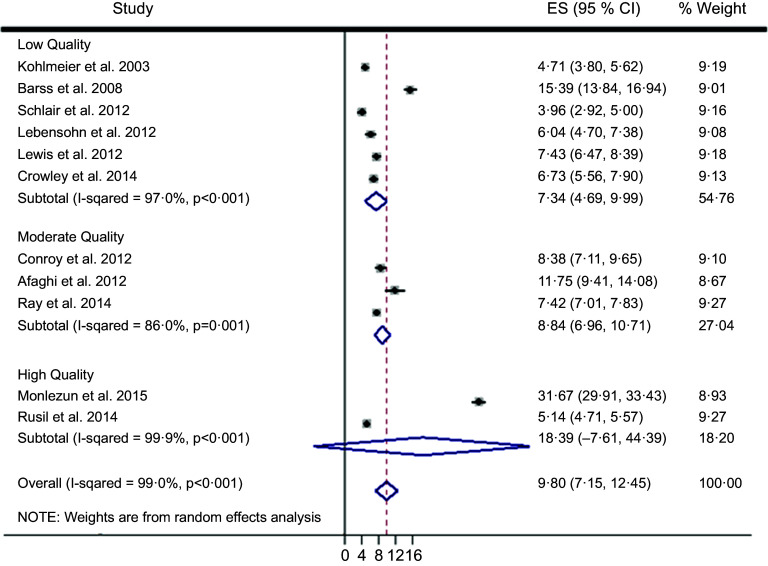
Machine learning-augmented meta-analysis of competency improvement with nutrition education by STROBE study quality

The search scheme was utilised to identify 212 citations of which eleven met study criteria (*n* 1698 trainees, Fig. 1)^(15–23,47,48)^. Random forest multiple imputation generated imputed values for missing data (NRMSE 0·103). Significant heterogeneity was noted among the included studies (*Q*-test *P* < 0·001; *I* ^2^ 99·0 %). Therefore, random effects meta-analysis was selected over fixed effects, and subsequently calculated an overall ES across two high, three moderate and six low-quality studies of 9·80 (95 % CI (7·15, 12·45), 95 % CI (6·87, 13·85); *P* < 0·001) (Fig. 2). The two high-quality studies as characterised by the STROBE criteria had a significantly higher ES than the moderate and low-quality studies (*Q*-test *P* < 0·001; *I* ^2^ 99·0 %). Nutrition education interventions among the high, moderate and low-quality studies improved medical student competencies counseling patients on nutrition (improvement %, pooled s d %) by 41 % (4 %), 18 % (13 %) and 21 (14 %). The largest ES was demonstrated by Monlezun *et al.* (31·67, 95 % CI (29·91, 33·43)) which was significantly greater than the other high-quality study ES (5·14, 95 % CI (4·71, 5·57)), with improved competencies (pooled s d) of 72 % (2 %) *v*. 9 % (5 %), respectively. The number needed to treat for the top performing study was 12.

**Fig. 3:**
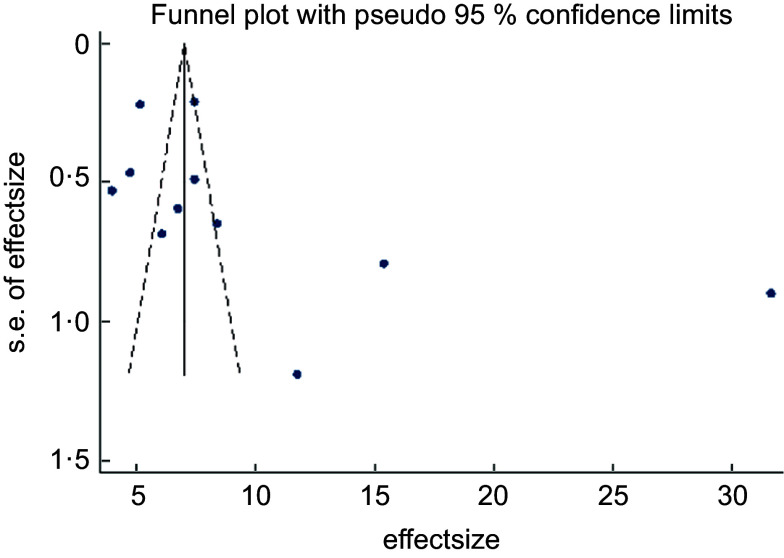
Publication bias with funnel plot (pseudo 95 % CI)

**Fig. 4:**
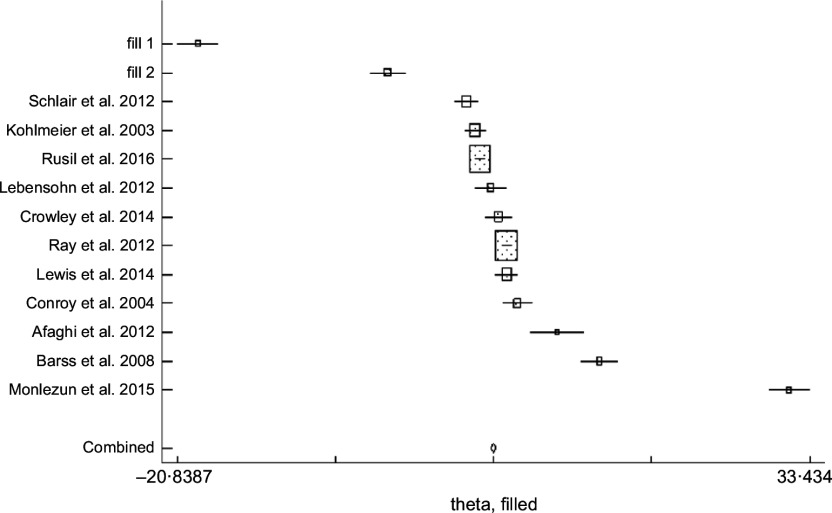
Bias adjustment with trim and fill

**Fig. 5:**
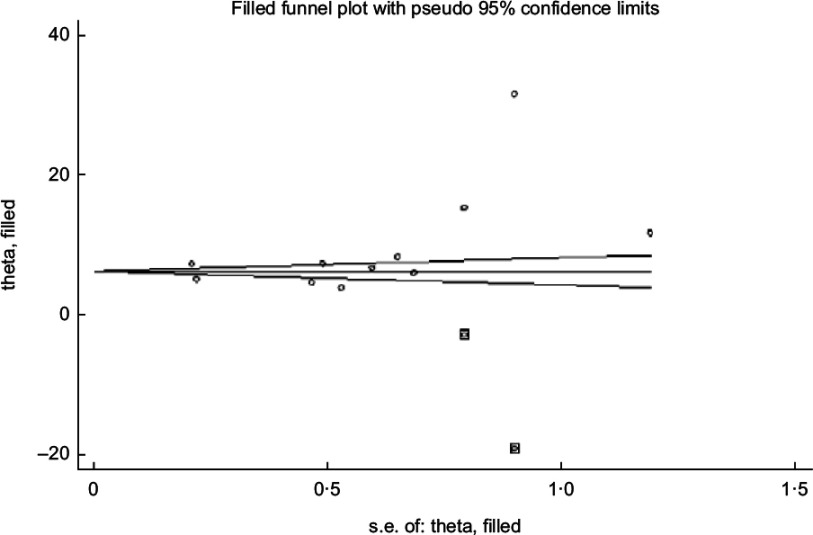
Bias control with trim and fill funnel plot (pseudo 95 % CI)

The asymmetrical funnel plot (Fig. 3) and the Harbord–Egger (*P* = 0·128) and Begg–Mazumdar (*P* = 0·020) statistical tests were not conclusive for non-significant small study effects or publication bias. To address this, trim and fill with random effects meta-analysis was applied to generate an adjusted overall ES of 6·66 (95 % CI (3·38, 9·94); *P* < 0·001) (Fig. 4) with a modified funnel plot that included two simulated studies that were quantitatively predicted to be omitted from the literature secondary to publication bias (Fig. 5).

The unadjusted and adjusted random effects meta-analysis results were then compared with the ML meta-analysis results. ML-linear regression (ML-LR) with 10-fold cross validation was the initial algorithm tested due to the outcome being continuous. It generated an overall ES of 9·81 (95 % CI (9·42, 10·20)), but the root relative squared error was optimised from 97·99 % to 81·31 % by instead using ML-LR with locally weighted learning (LWL), which integrated ML-LR with a specified weighted instances handler^(49,50)^. This algorithm was superior to the other 24, including reduced error pruning tree with backfitting, random tree, random forest, decision stump, zero-R, M5 model tree, decision table, input mapped classifier, weighted instances handler wrapper, vote, stacking, regression by discretisation, random sub-space, randomisable filtered classifier, random committee, multi-scheme, cross-validation parameter selection, bagging, attribute selected classifier, additive regression, K-nearest neighbours, support vector machine with regression, multi-layer perceptron with backward propagation and Gaussian regression.

### Part B: cohort study

**Fig. 6:**
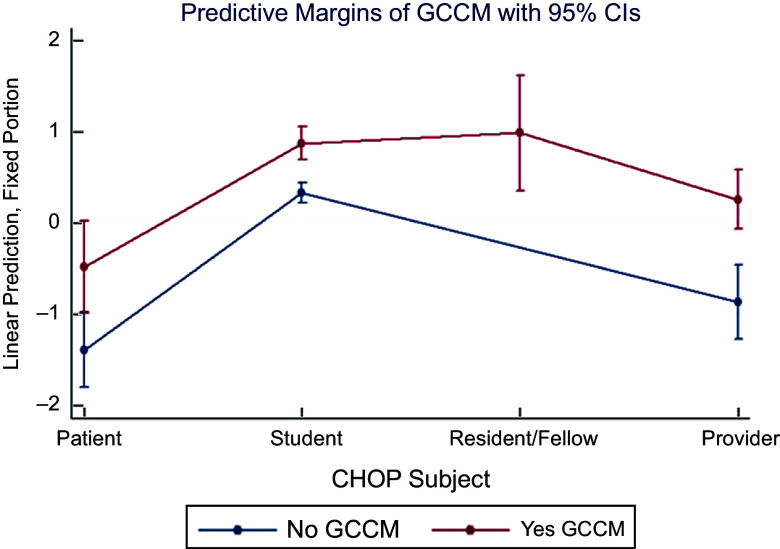
Machine learning-augmented multi-level mixed effects cohort analysis of hands-on cooking and nutrition education (GCCM) improving Mediterranean diet adherence*

The hands-on cooking and nutrition education curriculum (GCCM) from the top performing study was thus applied to CHOP. Fully adjusting for age, sex, race, special diet, institution and time invariant unobserved traits, GCCM compared with standard medical care and education significantly increased the odds of superior diets (high/medium *v*. low MedDiet adherence) for residents/fellows most (OR 10·79, 95 % CI (4·94, 23·58); *P* < 0·001) followed by healthcare students (OR 9·62, 95 % CI (5·92, 15·63); *P* < 0·001), providers (OR 5·19, 95 % CI (3·23, 8·32), *P* < 0·001) and patients (OR 2·48, 95 % CI (1·38, 4·45); *P* = 0·002) compared with patients without GCCM treatment, with results consistent with those from ML (Fig. 6).

## Discussion

The current study is the first known meta-analysis suggesting nutrition education improves medical trainee competencies providing patients dietary counseling for overall reduction and co-management of obesity and CVD (part A). It achieved this by applying a novel ML approach to traditional biostatistical random effects meta-analysis technique to produce more precise and efficient estimates of studies assessed using the standard STROBE criteria, with estimates adjusted using the well accepted trim and fill method. Additionally, the current study is the first to unite the following novel elements of a multi-centre prospective cohort study design assessing a hands-on cooking and nutrition education curriculum (informed by the above evidence-base) with ML augmentation of a longitudinal statistical analysis of medical trainees and providers, and thus demonstrating that such a curriculum improves their diets and therefore the diets of the patients they then taught (part B). Translating the clinical efficacy, cost-effectiveness and societal equity-enhancing aspects of public health nutrition into medicine globally may therefore may facilitated with this unique intervention that was united with the methodological advances of rigorous causal inference statistics integrated with ML (increasingly demonstrated to produce statistical results familiar to clinical audiences but with the added advantages of the greater versatility and efficiency of ML)^(51,52)^.

The clinical significance of these findings is they provide the first empirical codification supporting the eventual development of the evidence-based foundation of scalable nutrition education for obesity and nutrition-related chronic diseases including CVD, suggesting a blueprint for the next generation of medical professionals to ultimately reduce the explosive growth of these global healthcare challenges. Nutrition already has an accepted role reducing the health inequities and economic burden of obesity, CVD and other nutritionally related chronic disease epidemics^(2–4,53,54)^. By educating medical trainees about culinary medicine through hands-on cooking and nutrition education in teaching kitchens, an increasing number of randomised trials and causal inference analyses of large cohort studies are showing not only that trainees have superior mastery of core counseling topics but also that patients taught by such trainees in those kitchens subsequently have superior diets and health outcomes^(47,52–58)^. This programmatic advancement may better equip medical professionals to complement their pharmacological and surgical response to the obesity and nutrition-related chronic disease epidemics with their improved mastery of the social determinants of health. But the current analysis suggests that this advancement may be further accelerated through the systematic application of emerging ML techniques to allow more accurate, rapid, efficient and automated analyses of high-dimensional and heterogenous data in real-time, to guide quality improvement in medical trainee and patient education.

One such potentially valuable ML application in this field is the locally weighted regression that is memory based:




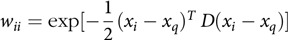




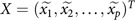




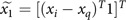




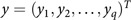




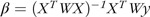




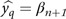




with 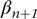 indicating regression vector 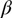 to the 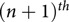 element, *X* is representing the matrix with vector *y*, *W* is the diagonal weight matrix and query point 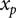 that has *p* training points 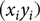 ^(59)^. The traditional statistical technique of the DerSimonian & Laird non-iterative random effects meta-analysis has several notable mathematical differences from the above approach. The DerSimonian & Laird technique historically is a popular random effects approach that has the advantage of comparable performance with less computational intensity *v*. competing methods dealing with study heterogeneity^(60–63)^. This method is described with 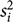 for the variance of *K* studies to produce an ES of 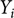 :

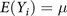




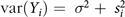




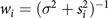




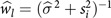




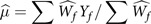




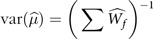




Despite the above strengths of this meta-analysis in both its theoretical, analytic and programmatic advances, it has multiple weaknesses. These include the wide variety of studies (even among the high-quality studies per STROBE criteria) ranging from the sample traits, type of nutrition education, the assessment tool, the outcomes, study duration, analytic approach and even the reporting. The current analysis further suffers from a lower number of studies and particularly high-quality studies standardised using the best practices in the above intervention and study traits. The random effects meta-analysis technique, STROBE criteria, trim and fill method and ML augmentation are meant to reduce the impact of weaknesses of the current analysis but cannot nullify its effects on study validity, and so these results should be interpreted with them in mind. The limitations of the above cohort study as the second phase of the current study include its non-randomised design assessing a voluntary curriculum.

Our results indicate that such varied education models may benefit from a more robust approach that draws from the hallmarks of evidence-based education and rigorous methodologies. One such promising approach is that utilised by the only study in the analysis to attain the STROBE classification of a high-quality study and have a significant ES that of GCCM^(49)^. The current study utilised control comparison, validated survey metrics, multi-year longitudinal follow-up, deliberate practice counseling patients, adequately powered sample size, statistical methodologies such as fixed effects regression to allow causal inference controlling for confounders and the MedDiet. The study is part of the overall CHOP cohort as the world’s first study of hands-on cooking and nutrition education for medical trainees, providers and patients, run through Tulane University School of Medicine’s GCCM. Since its launch up to May 2017, GCCM has provided 24 680+ hours of hands-on cooking and nutrition education to 4051+ medical students, physicians and patients across forty-five medical schools, hospitals and colleges.

Such interventions may be increasingly useful as health systems internationally are progressively outmatched by the rising clinical, cost and inequity toll of obesity and its nutrition-related chronic disease epidemics. Culinary medicine as preventive obesity management may be a promising scalable intervention long term through better trained medical professionals. But the benefit may also be short term through better educated patients served in hands-on cooking and nutrition education classes, led by the same trainees who first were educated themselves in those classes. The insistence on high-quality studies using rigorous, novel integration of traditional statistics and artificial intelligence-driven ML may help accelerate the evidence base for such healthcare innovations as culinary medicine uniting medicine and public health through population health management. The current study attempts to fill such theoretical, methodological and programmatic gaps in the associated research fields, for the equitable and cost effective good of patients globally particularly with obesity and its related complications.
